# Free-space propagation of high-dimensional structured optical fields in an urban environment

**DOI:** 10.1126/sciadv.1700552

**Published:** 2017-10-25

**Authors:** Martin P. J. Lavery, Christian Peuntinger, Kevin Günthner, Peter Banzer, Dominique Elser, Robert W. Boyd, Miles J. Padgett, Christoph Marquardt, Gerd Leuchs

**Affiliations:** 1 School of Engineering and School of Physics, University of Glasgow, Glasgow, UK.; 2 Max Planck Institute for the Science of Light and Institute of Optics, Information and Photonic, Friedrich-Alexander-Universität Erlangen-Nürnberg, Erlangen, Germany.; 3 Department of Physics, University of Otago, Dunedin, New Zealand.; 4 Max Planck–University of Ottawa Centre for Extreme and Quantum Photonics, 25 Templeton Street, Ottawa, Ontario K1N 6N5, Canada.; 5 Institute of Optics, University of Rochester, Rochester, NY 14627, USA.; 6 Department of Physics, University of Ottawa, Ottawa, Ontario, Canada.

## Abstract

Spatially structured optical fields have been used to enhance the functionality of a wide variety of systems that use light for sensing or information transfer. As higher-dimensional modes become a solution of choice in optical systems, it is important to develop channel models that suitably predict the effect of atmospheric turbulence on these modes. We investigate the propagation of a set of orthogonal spatial modes across a free-space channel between two buildings separated by 1.6 km. Given the circular geometry of a common optical lens, the orthogonal mode set we choose to implement is that described by the Laguerre-Gaussian (LG) field equations. Our study focuses on the preservation of phase purity, which is vital for spatial multiplexing and any system requiring full quantum-state tomography. We present experimental data for the modal degradation in a real urban environment and draw a comparison to recognized theoretical predictions of the link. Our findings indicate that adaptations to channel models are required to simulate the effects of atmospheric turbulence placed on high-dimensional structured modes that propagate over a long distance. Our study indicates that with mitigation of vortex splitting, potentially through precorrection techniques, one could overcome the challenges in a real point-to-point free-space channel in an urban environment.

## Teaser

This study of structured light’s propagation across a 1.6-km free-space link indicates that adaptations to models may be required.

## INTRODUCTION

In recent years, the study of spatially structured optical field has led to many impressive demonstrations in stimulated emission depletion microscopy (*1*), optical trapping (*2*, *3*), remote sensing (*4*), imaging systems (*5*, *6*), light detection and ranging (*7*), communication systems (*8*, *9*), and quantum information (*10*–*12*). As these technologies mature, an understanding of the hurdles facing deployment will become fundamentally important. The propagation of spatially structured optical fields in free space is regarded as a leading challenge for many sensing and communication systems (*13*). A key issue in extending the range of these systems is the modal degradation that occurs during propagation through atmospheric turbulence. The time-dependent and random variations in temperature and pressure of the atmosphere result in a change in optical density of the atmosphere (*14*). This results in a spatially dependent change of the refractive index, leading to a phase distortion across a transmitted beam (*15*–*17*). Many current models for free-space transmission of optical fields date back to the work of David L. Fried, Andrey Kolmogorov, and their contemporaries in the mid-20th century (*18*). These models have proved solid for planar wavefronts, such as those used in astronomy (*19*). However, as higher-dimensional modes potentially become a solution of choice in optical systems, it is important to develop channel models that suitably predict the effects of atmospheric turbulence on these spatially structured optical fields.

A sector currently driving many of the developments in structured photonics is the communication systems sector, where many forms of spatial encoding have been demonstrated (*8*, *20*–*24*). The appropriate way one segments the spatial degree of freedom is related to specifics of the free-space system (*13*). Given the circular geometry of a common optical lens, the orthogonal mode set we choose to implement is that described by the Laguerre-Gaussian (LG) field equations, which are characterized by orthogonal eigenvalues ℓ and *p*, corresponding to the azimuthal and radial components, respectively. LG modes have received notoriety for the orbital angular momentum (OAM) that is carried by their helical wavefronts (*25*). Beams with a transverse amplitude profile of A(r)exp(iℓφ) carry an OAM of ℓℏ per photon, with *r* and φ as the radial and angular coordinates, respectively (*25*). We study the measured optical field degradation that is induced by the temperature and pressure variations in the atmosphere as the light propagates across the channel. Our study focuses on the preservation of phase purity, which is vital for spatial multiplexing and any system requiring full quantum-state tomography. Previous work on studying this OAM cross-talk has been considered within the thin-phase regime, where the longitudinally varying phase retardance arising from the local changes in optical density can be approximated as a perturbing spatial phase profile in a single plane. This phase profile can be readily emulated through holographic techniques, or other benchtop apparatus, to replicate the condition that light propagating over a link may experience (*15*–*17*). A concern not discussed in these atmospheric turbulence studies is the structural stability of OAM modes with ℓ>1 that are perturbed by a phase aberration. It has been previously documented that, in the presence of any weak noncylindrically symmetric aberration, a high-order vortex of index ℓ will break up to give ℓ individual vortices of index 1 upon propagation (*26*–*29*).

It has been shown that the thin-phase approximation is only valid for short free-space link lengths and not suitable to study the degree of channel degradation that would occur over longer or more turbulent links (*15*, *16*). For the implementation of last-mile free-space optical links, it is important to understand the challenges that atmospheric tip-tilt and higher-order turbulence effects place on the design of a deployable system. A recent demonstration by Krenn *et al.* (*30*, *31*) has indicated that the spatial intensity modulation of a laser mode is preserved over long-distance propagation at distances of up to 143 km. Inspired by the impressive resilience of the intensity structure, an important question is whether the phase profile of these modes is preserved over long-distance propagation. The preservation of the phase structure is vital for space-division multiplexing (SDM) schemes and systems that require a full-state measurement at the receiver, such as quantum key distribution (QKD).

**Fig. 1:**
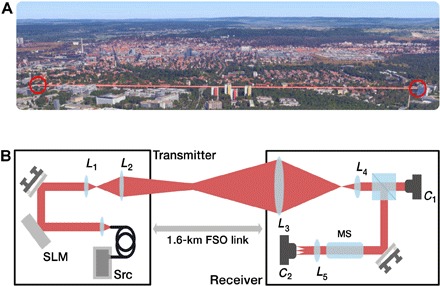
Experimental setup. (**A**) Our 1.6-km free-space link is over the city of Erlangen in Germany. The optical beam is transmitted over both buildings, roads, and wooded areas as pictured. Image is courtesy of Google Map data 2016. (**B**) A fiber-coupled optical linearly polarized source with a wavelength of 809 nm is collimated and illuminates a spatial light modulator (SLM). An ℓ-forked hologram is displayed on the surface of the SLM. This diffractive hologram imparts the desired azimuthally varying phase pattern onto the wavefront of the incident optical beam. The beam is then magnified by a telescope (consisting of two lenses, *L*_1_ and *L*_2_) to give a maximum diameter of approximately 40 mm. After propagation over the 1.6-km free-space optical link, the light is collected by a receiver lens with a diameter of 150 mm and a focal length of 800 mm (*L*_3_). *L*_4_ is used to demagnify the beam to approximately 10 mm in diameter, allowing the analysis of the received light. A beam splitter is used to simultaneously image the received beams on a camera, *C*_1_, and pass the light through an OAM mode sorter (MS) to measure the modal content of the received light (*C*_2_).

Here, we present a study of the degradation in channel fidelity that occurs in nine particular OAM modes (ℓ=0,±1,±2,±3, and ±4) propagated over a 1.6-km free-space link above the city of Erlangen in Germany (Fig. 1A) (*32*, *33*). We report the effects resulting from the atmospheric tip-tilt and higher-order aberrations, as wells as impure modal generation, on the channel cross-talk. Our results indicate that current atmospheric turbulence models do not adequately predict the degradation expected by spatially structured optical fields, where it is observed that vortices with ℓ>1 split upon propagation over our free-space channel. The observed vortex splitting resulted in a change of the measured average OAM of the received beam. This indicates that adaptive optical techniques will be required to mitigate the effects of turbulence.

In our system, the aperture is approximately 50% of the beam diameter for ℓ=4 (*34*). Given the restrictions on the system aperture size, we limited the experiment to considering the orthogonal azimuthal integer ℓ because orthogonal radial integers of *P* > 0 were not uniformly collected efficiently by our aperture for all selected ℓ values. To maximize the collection efficiency, our link is designed to have a focal plane 800 m from the transmitter. This link has a calculated Fresnel number of *F* = 8.69 and a spatial resolution of approximately 5 mm, confirming that our receiver telescope is not in the far field of the transmitter. We further consider the propagation of superposition modes over the link, allowing a direct comparison to the recent work by Krenn *et al.* (*30*), which demonstrated the resilience of the spatial intensity structure even over long-distance propagation. Our results independently confirm that the intensity structure is maintained similar to that of an imaging system; however, the phase information is considerably distorted because of the optical aberrations induced by the atmospheric turbulence.

## RESULTS

**Fig. 2:**
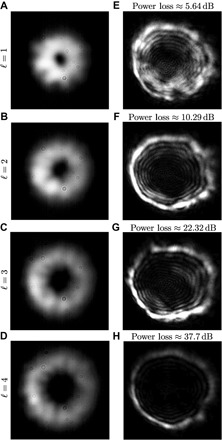
Images of the optical field at transmitter and receiver. (**A** to **D**) Image of the generated modes at the transmitter telescope. (**E** to **H**) Images of the received beam with ℓ=1,2,3, and 4 captured by a camera, *C*_1_, in Fig. 1B, respectively. As the ℓ of the transmitted mode increases, the beam diameter of the optical mode also increases. As the receiver lens has a fixed diameter, this increase in beam waist results in power loss that increases with ℓ. The measured power loss is the difference between the transmitted power, 0.75 mW, and the respective received power at the receiver measured by *C*_1_. The power loss (and that expected from the theoretical divergence of the beam) was measured to be 5.64 dB (0.043 dB), 10.29 dB (1.14 dB), 22.23 dB (6.70 dB), and 37.3 dB (17.11 dB), respectively.

The tip-tilt aberration arising from atmospheric turbulence and mechanical movement is one of the largest concerns within a free-space optical system. This form of atmospheric aberration results in a change of the beam propagation direction between a transmitter and a receiver. One can characterize the degree of tip-tilt by measuring the centroid location, which we will call the center of mass (CofM), of a received Gaussian beam. We measure the CofM over 20 s, sampling at 8-ms intervals. Under particular configuration and atmospherical conditions, we observe an average radial variation of 18 mm from the center (see Fig. 2A). This tip-tilt data can be used to predict the expected OAM mode cross-talk one would expect to measure for a particular input. A comparison between this expected cross-talk and the experimentally measured cross-talk can give an indication of the contribution of tip-tilt aberrations. It should be noted that no automated correction is used to reduce the effects of tip-tilt aberrations.

**Fig. 3:**
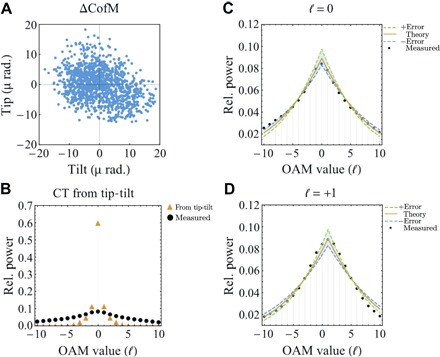
Tip-tilt and thin-phase turbulence model comparison. (**A**) CofM variation from an on-axis position resulting from tip-tilt atmospheric turbulence measured over 20 s. (**B**) To analyze the contribution of tip-tilt atmospheric turbulence, the CofM tracking data were used to simulate the expected channel cross-talk arising solely from this tip-tilt variation, which is overplotted with the experimental measured OAM spectrum. It can be seen that these two data sets do not match, indicating that turbulence beyond solely tip-tilt is affecting the optical beam as it propagates over the link. (**C** and **D**) Averaged broadened OAM spectrum measured for an ℓ=0 and ℓ=1 mode transmitted over the 1.6-km link, overplotted with the expected spectrum arising from thin-phase turbulence. Dashed lines represent the error in the measurement of *D*/*r*_0_.

The measured frame-by-frame CofM data are used to generate simulated “misaligned” pure OAM states for each recorded frame. The misaligned OAM state is predicted by altering the beam axis to the CofM location, along with assuming that the phase variation across the beam waist is angled at $\theta_{y}={\text{tan}}^{-1}\frac{\delta y}{L}$ and $\theta_{x}={\text{tan}}^{-1}\frac{\delta x}{L}$ for tip and tilt, respectively, where *L* is the length of the free-space link. This set of spectra is then summed and averaged to give an expected modal cross-talk arising solely from tip-tilt aberrations. To compare the modeled spectrum solely arising from tip-tilt aberrations with the experimentally measured OAM spectrum, both results are co-plotted in Fig. 3B. It can be seen that the results do not match the investigated scenario, which indicates the additional contribution from other optical aberrations.

Tip-tilt aberrations are generally the most commonly considered atmospheric turbulence effect; however, higher-order aberrations can be present within long-distance free-space links. To analyze the effects of higher-order optical aberrations in a free-space link, one can adopt the Fried parameter *r*_0_, which is a measure of the transverse distance scale over which the refractive index is correlated (*14*). To characterize the effect of turbulence on the optical system, the *D*/*r*_0_ ratio is considered, where *D* is the aperture of the system. This ratio sets two limiting cases. First, when *D*/*r*_0_ < 1, the resolution of the system is limited by its aperture. Second, in the case of *D*/*r*_0_ > 1, the atmosphere limits the system’s ability to resolve an object (*14*).

A common method to determine the Fried parameter, *r*_0_, is through a seeing disc measurement. This is achieved through focusing and characterization of the full width at half maximum (FWHM) of the point spread function of an ℓ=0 mode intensity (that is, fundamental Gaussian mode) at the receiver. This characterization allows determination of the degree of atmospheric turbulence that the propagated beams experienced, where $r_{0}\approx \frac{\lambda }{\text{FWHM}}$. In our experimental realization, the typical strength of atmospheric turbulence is hereby determined as *D*/*r*_0_ = 7.62 ± 1.04, where *D* is the aperture size of 150 mm.

The OAM modal cross-talk can be calculated by considering Kolmogorov statistical models of atmospheric turbulence (*35*). Considering a single OAM mode, $\psi_{\ell }$, transmitted through an ensemble average of many turbulent phase screens, the power *s*_Δ_ in a particular mode, $\psi_{\ell +\Delta }$, is given by

$$s_{\Delta }=\frac{1}{\pi }\int_{0}^{1}\frac{2r}{D}dr\int_{0}^{2\pi }d\theta e^{-3.44{\left[(\frac{2r}{r_{0}})(\text{sin}\frac{\theta }{2})\right]}^{5/3}}\text{cos}\Delta \theta$$

where Δ is an integer step in the mode index ℓ=|1| (*16*).

In our experiments, beams carrying OAM with ±1, ±2, ±3, and ±4 were individually generated and then propagated over the link (Fig. 1B). At the receiver, the cross-talk between channels was measured. This measured channel cross-talk was compared with the theoretical OAM spectra that correspond to the measured atmospheric turbulence strength across the link. The modeling was found to fit with the results obtained for ℓ=0 (Fig. 3B) and ℓ=±1 (ℓ=1 is shown in Fig. 3D). In the thin-phase turbulence model, one would expect the measured modal spectrum to have a mean value approximately equal to the ℓ of the transmitted mode. These turbulence models are commonly applied to astronomical systems where the light is propagating through an atmospherically neutral transmission path until it encounters atmospheric turbulence close to the receiver aperture. This form of turbulence can be mitigated through single-plane adaptive optical systems.

**Fig. 4:**
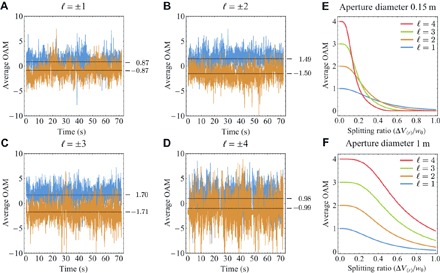
Observation of change in average OAM value. (**A** to **D**) Mean OAM value for the measured OAM spectrum for each recorded frame over 70 s, with a frame rate of 120 frames/s. It is observed that the average value of OAM measured, marked as a black dashed line, at the receiver does not match the transmitted mode. (**E** and **F**) Simulation of the measured average value of OAM as a function of vortex-splitting ratio for the cases where the transmitter is restricting the collection of the optical field and for the case where the beam is completely collected by a 1-m receiver aperture.

However, point-to-point transmission systems on earth are usually not of this form. Here, bulk (thick) optical turbulence must be considered. In transmission paths experiencing thick atmospheric turbulence, one could represent the link as several turbulent phase screens, each separated by some distance of propagation, which would result in increased degradation of the optical wavefront (*36*). Previous studies have indicated that one would expect the received OAM spectrum to be centered at the transmitted mode order, similar to that seen in thin atmospheric turbulence just with a different spectral width (*36*). However, our experimental results indicated that this does not hold for |ℓ|>1, where the mean of the OAM spectrum was less than the ℓ of the transmitted mode (Fig. 4, A to D).

Because higher-order OAM modes have a larger effective beam waist compared to those with lower values of ℓ, less power is received from the high-intensity annulus (Fig. 2) (*37*). Hence, the received field of view encompasses more of the dark region in the center of the spatial mode than of the high-intensity annulus. This decrease in received power from the transmitted mode can potentially lead to other modal components becoming more dominant in the received OAM spectra, changing the average OAM received.

In the presence of mild turbulence or any weak noncylindrically symmetric aberration, a high-order vortex of index ℓ will break up to give ℓ individual vortices of index 1 upon propagation (Fig. 4) (*26*–*29*). Hence, we simulate the expected result for cases were vortex splitting occurs during the propagation of an optical mode over the optical link. Using a plane-wave decomposition model, we only considered diffraction effects and boundary conditions imposed on light collected by a fixed-size receiver aperture. Because we wish to only consider the contribution of vortex splitting, we do not implement any turbulence model within our numerical simulation. A vortex-splitting ratio was defined as $V=\frac{\Delta V_{<r>}}{w_{0}}$, where Δ*V*_<r>_ is the average radial distance from the beam origin for the individual vortices and *w*_0_ is the beam waist of the transmitted mode. The expected average OAM was determined for two cases: first, where no bounding aperture is impeding the propagation beam, and second, where the 150-mm aperture of our experimental system is imposed, as shown in Fig. 4, E and F, respectively. In both cases, there is an expected change in average OAM; however, the effect is amplified in the presence of a bounding aperture that is smaller than the annulus of the optical mode.

Therefore, our experimental results strongly indicate the presence of vortex splitting. This splitting results in an offset in average OAM measured after propagation over the link and corresponds to a received ratio of approximately *V* = 0.18 for ℓ=±3 and *V* = 0.2 for ℓ=±4. This result also indicates that higher-order modes are more sensitive to the effects of weak noncylindrically symmetric aberration than lower-order modes. This vortex splitting could arise from system back-reflections, static optical aberrations, or scattered light in the preparation of the optical mode. In addition, atmospheric turbulence near the transmitter that perturbs the mode under-propagation could result in vortex splitting, hence changing the measured average OAM.

From a system modeling that matches our experimental parameters, for an ℓ=4 mode to be received with a mode purity of >99% (similar to that required for some simple modulation scheme, such as quadrature phase-shift keying), we will require a vortex-splitting ratio at the transmitter with *V* < 0.03. This places stringent requirements on the modes one uses and the aperture size chosen, when one designs a system for deployment over a long-distance link.

### Superposition of OAM modes

**Fig. 5:**
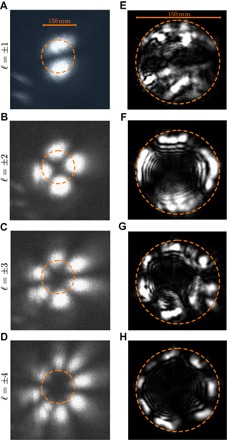
Superpositions of OAM. Superpositions have previously been shown to propagate over long turbulent optical paths, without drastic degradation of their intensity profile. (**A** to **D**) To assess this situation in our 1.6-km link, superpositions of ℓ=±1,±2,±3, and ±4 were generated and propagated over the optical link. The superposition states were projected on a large white screen, and the scattered light was imaged by a camera. (**E** to **H**) The same modes were collected by the 150-mm receiver aperture; however, the structure is truncated due to the restricted aperture size, as indicated by the dashed circles overlaid on each image.

Experimental demonstrations of the resilience of intensity patterns arising from the superposition of two equal and opposite OAM modes have been shown over 3 and 160 km, respectively (*26*, *27*). The superposition imprints a distinctive pattern of 2ℓ petals arranged around the annulus of the mode (*38*–*40*). These modes have a binary phase profile, azimuthally stepping between 0 and π. Similar to an imaging system, the intensity has been shown to be maintained at the receiver. To make a direct comparison of our link to the result previously shown by Krenn *et al.* (*30*), superpositions of ℓ=±1,±2,±3, and ± 4 are generated and the intensity profile is imaged at the receiver (Fig. 5, A to D). As expected, the intensity profile is recognizable as a set of 2ℓ petals. When imaged through the receiver aperture, depicted as an orange circle in Fig. 5, the modes have considerable amounts of aberration in the high-intensity regions, indicating higher-order atmospheric turbulence effects. Although the intensity profile seems to maintain its structure, the phase has experienced aberration due to propagation through the turbulent link. These aberrations will result in considerable cross-talk when one wishes to implement a system that requires phase-sensitive measurements, such as spatial demodulation techniques and QKD systems.

### Mitigation of turbulence

**Fig. 6:**
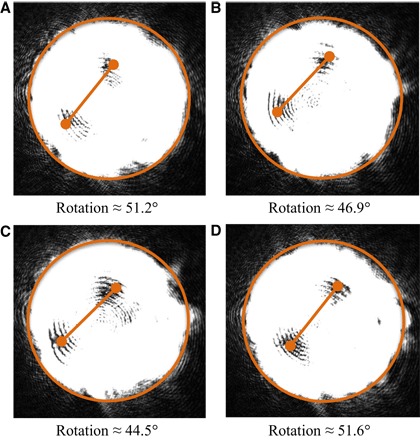
Change in vortex-splitting ratio. Modal purity is an important consideration for the long-distance transmission of spatial modes for use in communications. To enhance our determination of a change in purity, an *ℓ* = 0 component, with a 30% weighting, was added to an *ℓ* = 2 mode. This yields a vortex-splitting ratio of *V* = 0.4. By saturating the CCD, we can locate the intensity nulls that indicate the position of the vortices in the beam. (**A** to **D**) Four samples are shown, where each recorded incidence is respectively separated in time by 0.08 s. Overlaid is a fixed-size bar, indicating the expected vortex separation from a system simulation. It can be seen from frame to frame that there is a small but noticeable change in the separation and relative position of the vortex locations. This change in relative position and separation indicates that dynamic variations in the free-space channel are causing a variation in the vortex splitting observed.

Because our results indicate that although the modal superpositions maintain their spatial intensity structure, their spatial phase profile is subject to significant aberrations during propagation through the atmosphere. For one to use these spatial modes for information transfer, mitigation techniques will be needed. From theory, we find that, for ℓ=0 and ℓ=±1, the data closely agree with those expected from the thin-phase turbulence model, and therefore, one could potentially implement a traditional adaptive optical system comprising phase correction applied to a single plane. However, the situation for |ℓ|>1 may require a more complex form of mitigation that deals with the vortex splitting that arises from the turbulence. It is important to determine whether the source of vortex splitting is entirely from fixed system aberrations or whether the continually varying atmospheric turbulence is contributing to the measured 6% error. To determine whether there is a change in the modal purity on the time scale of the turbulent atmosphere, a weighted superposition of 70% ℓ=2 and 30% ℓ=0 was propagated over the link. This superposition results in spatially separated vortex poles that can be tracked in time (Fig. 6, A to D). For static aberrations in the optical system, the relative position of the two separated vortices would remain constant. It should be noted that global movement of the pair is expected because the beam is known to experience tip-tilt aberrations. However, it is observed that there are relative shifts in both vortex locations and relative angular position varying faster than 0.08 s, indicating that the varying atmosphere is contributing to the degradation in modal purity. Although it appears more challenging, we feel that further experimental investigation of appropriate mitigation techniques could potentially overcome the observed modal degradation.

## DISCUSSION

Here, we set out to measure the effects on both the phase and intensity of OAM modes over a real link in an urban environment to assess the viability of OAM for use in SDM and for quantum information transfer. Our free-space link was 1.6 km in length, where our optical channel passed over fields and streets and close to high-rise buildings. We observed that OAM mode superpositions maintain their intensity structure, confirming the results presented by Krenn *et al*. (*30*). However, for many applications, phase retrieval, such as high-bandwidth SDM and quantum information transfer using the full optical topology of a laser mode, is required. Using passive OAM mode-sorting optics, simultaneous measurement of the channel cross-talk over many modes was recorded. The phase aberrations arising from the turbulent atmosphere resulted in considerable cross-talk, resulting in a broad OAM spectrum. When compared to the cross-talk expected from thin-phase turbulence models, we find that, for low-order OAM modes, the measured cross-talk fits closely to theory. For higher-order modes, this was not the case, where the expectation value did not correspond to the sent OAM mode. By considering the change in beam size from the transmitter to the receiver after 1.6 km of propagation, these results are expected in the case where the vortex splitting occurs before collection at the receiver. Our results indicate that with careful modal generation, where vortex-splitting ratio is *V* < 0.03, and inclusion of single-plane atmospheric turbulence mitigation techniques, we expect that a link deployed in an urban environment could be functional for both communication and remote sensing systems.

## MATERIALS AND METHODS

### Experimental setup

The OAM mode transmitter comprises a diode laser source expanded to illuminating an SLM, which is encoded with an ℓ-forked hologram to generate the required, in our case linearly polarized, OAM mode (Fig. 1). These modes are then further expanded using a telescope to have an approximate beam size of 40 mm. This beam is propagated over the 1.6-km-long free-space link (Fig. 1). The modal receiver comprises a collection lens with an aperture of 150 mm diameter and a focal length of 800 mm. A power loss was measured at the receiver aperture of approximately 5.64 dB of the incoming light with ℓ=1. A second lens is included in the telescope to demagnify the collected light beam to have a diameter of approximately 10 mm. To detect the OAM content and, hence, also the cross-talk between OAM channels, a device known as a mode sorter is placed at the output of the demagnification telescope. The mode sorter uses two refractive elements, which transform OAM states into transverse momentum states (that is, tilted plane waves) (*41*, *42*). These elements transform a beam of the form exp(iℓφ) to exp(iℓax), where *a* is a scaling parameter. A lens is used to focus these transformed states to discrete spots at a charge-coupled device (CCD) camera placed in its focal plane. Adjacent equally sized regions are defined within the measured CCD image, with each region corresponding to a specific OAM mode. The sum of the measured pixel values in each of these regions is proportional to the power of the beam in each OAM mode (*42*).

### Numerical modeling of predicted cross-talk arising from tip-tilt aberrations

Pure OAM states are only single-mode states with respect to one specific axis. This axis is the *z* axis of the cylindrical polar coordinate system in which the complex amplitude cross section in a transverse plane (*z* = constant) of a pure mode with a specific ℓ value can be written in the form exp(iℓθ). We call this axis the beam axis. When described with respect to a different axis, that is, the measurement axis, a single OAM state becomes a superposition of a number of states (*43*, *44*). As the LG modes form an orthonormal basis, any beam cross section ψ(*x*, *y*) can be written, using the bra-ket notation [$\Psi_{\ell ,p}(x,y)=|\ell p\rangle$, |ψ〉 = ψ(*x*, *y*)], where $\Psi_{\ell ,p}$ is the complex amplitude of an LG mode whose beam axis coincides with the measurement axis, in the form

$$|\psi \rangle =\sum_{\ell =-\infty }^{\infty }\sum_{p=0}^{\infty }|\ell p\rangle \langle \ell p|\psi \rangle$$

The power in the component |ℓp〉 is then given by the modulus squared of the coefficient for that component, namely

$$P_{\ell ,p}={\left|\langle \ell p|\psi \rangle \right|}^{2}$$

The power in all components with the same value of ℓ is given by

$$P_{\ell }=\sum_{P=0}^{\infty }P_{\ell ,p}=\sum_{P=0}^{\infty }{\left|\langle \ell p|\psi \rangle \right|}^{2}$$

The set of these powers $P_{\ell }$ is the OAM spectrum we expect to measure.

Our misaligned pure OAM states are also LG modes. The misalignment of the beam axis with respect to the measurement axis is described by the parameters Δ*x*, Δ*y*, tilt (α), and tip (β). The complex amplitude of this misaligned LG beam is

$$\psi (x,y)=\Psi_{\ell ,p}(x-\Delta x,y-\Delta y)\text{exp}[i\frac{2\pi }{\lambda }(x\text{sin}\alpha +y\text{sin}\beta )]$$

where *k* = 2π/λ, and λ is the chosen wavelength (*43*).
